# Cerebellar Hemorrhage Masquerading as Unilateral Vestibulopathy: A Case Report

**DOI:** 10.1155/crnm/9611619

**Published:** 2025-05-08

**Authors:** Thomas Zeyen, Thomas Klockgether, Christina Schaub, Daniel Paech, Timo Vogt, Delia Kurzwelly

**Affiliations:** ^1^Department of Neurology, University Hospital Bonn, Bonn, Germany; ^2^Department of Neuroradiology, University Hospital Bonn, Bonn, Germany; ^3^Department of Otorhinolaryngology, University Medical Center Bonn (UKB), Bonn, Germany

## Abstract

Pseudovestibular syndrome refers to central pathologies that mimic acute unilateral peripheral vestibulopathy, often posing a diagnostic challenge, particularly when key symptoms indicating a central origin are absent. The most common etiology is brain ischemia resulting from posterior inferior cerebellar artery occlusion. This article presents a rare case of a left paramedian cerebellar hemorrhage initially misdiagnosed as right-sided vestibular neuritis. Cerebellar hemorrhage can induce pseudovestibular syndrome by disrupting the connective fibers from the flocculus to the ipsilateral vestibular nucleus in the pons. Additionally, central pathologies affecting the vestibular system may occasionally manifest a pathological vestibulo-ocular reflex. This case report underscores the importance of considering potentially severe central-origin conditions in the differential diagnosis of seemingly benign unilateral peripheral vestibulopathy.

## 1. Introduction

Acute unilateral peripheral vestibulopathy leads to vestibular syndrome, characterized by rotational vertigo, nausea, vomiting, spontaneous horizontal nystagmus, and a tendency to fall toward the affected side [1, 2]. Notably, vestibular syndrome can be mimicked by central lesions affecting the neural connection between the cerebellar flocculus and the ipsilateral vestibular nucleus (VN). This condition is often caused by medial cerebellar infarction resulting from thrombotic occlusion of the posterior inferior cerebellar artery (PICA) or the anterior inferior cerebellar artery (AICA) and has been referred to as pseudovestibular syndrome [3].

Central lesions typically do not produce a pathological head impulse test (HIT), and the vestibulo-ocular reflex (VOR) examination remains normal. However, as reported by Park et al. [4, 5], an extremely rare isolated floccular infarction can impair the VOR, manifesting as spontaneous horizontal nystagmus toward the lesion side. In addition to ischemic lesions, pseudovestibular syndrome may also result from demyelinating lesions, as seen in multiple sclerosis, or from brain tumors. Additionally, there are various possible lesion localizations within the brainstem or cerebellum that can cause isolated vestibular syndrome [6, 7].

We here present a rare case of cerebellar hemorrhage causing pseudovestibular syndrome. This case highlights the continued diagnostic challenge of distinguishing peripheral vestibular disorders from central nervous system conditions, even when using well-established examination techniques such as Head Impulse, Nystagmus, Test of Skew (HINTS).

## 2. Case

An approximately 70-year-old patient presented to the emergency room in the morning with subacute onset of spinning vertigo and spontaneous horizontal nystagmus to the left. Mild, nonspecific dizziness had started the evening before, followed by progressive spinning vertigo and nausea during the night. On neurological examination, Grade II spontaneous horizontal nystagmus to the left was observed, without any vertical component. Then, a trunk tilt with consistent tendency to fall to the right was observed, but no other cranial nerve abnormalities were detected, especially oculomotor function was normal and skew deviation was not observed. There were no signs of paresis or sensory deficits, and deep tendon reflexes were intact. The nose–finger and heel–shin–slide tests were slightly dysmetric bilaterally, without clear lateralization. Due to the mild presentation and symmetry, it was not interpreted as clear evidence for cerebellar ataxia. Additionally, mild dysarthrophonia was observed, though without typical cerebellar speech dysfunction. The patient reported a history of slurred speech following partial resection of the right jaw due to parotid carcinoma.

**Figure 1:**
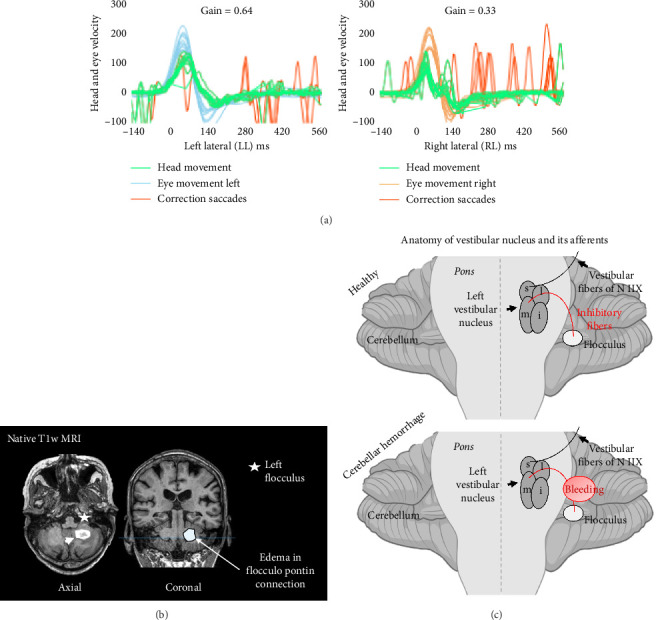
(a) Test results of the video-oculography–based HIT (vHIT) are shown. The examination demonstrates numerous catch-up saccades when stimulating the right lateral canal. Measurement revealed a VOR gain reduction on both sides, but more accentuated on the right (gain = 0.33). (b) Native T1-weighted MRI demonstrates hyperintense cerebellar hemorrhage in the left cerebellar lobe and slight perifocal edema affecting the flocculopontin connection. (c) The inhibitory connection between cerebellar flocculus and the ipsilateral vestibular nucleus and its affection by cerebellar hemorrhage. Abbreviations: i = inferior; l = lateral; m = medial; s = superior. Illustration was made using BioRender (BioRender.com, active academic individual license, created in BioRender. Zeyen, T. (2025) https://BioRender.com/467yr6a).

Initially, the patient was admitted to the otorhinolaryngology department with a tentative diagnosis of right-sided acute peripheral vestibulopathy. Video HIT (vHIT) revealed numerous catch-up saccades and a VOR gain reduction when stimulating the right lateral canal (gain = 0.33; Figure 1(a)), consistent with the suspected diagnosis. However, left VOR gain was also slightly reduced (gain = 0.64; Figure 1(a)). Given the patient's history of parotid carcinoma, computed tomography (CT) of the head was performed, revealing hemorrhage in the left cerebellar hemisphere. The patient was subsequently transferred to our stroke unit for further monitoring. Caloric testing was not conducted, as neuroimaging had already confirmed a central origin of the symptoms. Seven days after admission, magnetic resonance imaging (MRI) confirmed the cerebellar hemorrhage affecting the left posterior lobe (Figure 1(b)). The exact etiology could not be definitively determined. However, the patient had a history of hypertension, and MRI did not show signs of disseminated microbleeds, which are typical of cerebral amyloid angiopathy. No evidence of brain metastasis was found.

A few days after admission, the patient demonstrated rapid improvement. The treatment followed established guidelines for managing intracranial hemorrhage, including vigilant clinical monitoring at the local stroke unit and blood pressure management using oral medications. A regimen of ramipril (10 mg daily) and amlodipine (10 mg daily) was initiated during hospitalization and continued after discharge, aligning with national guidelines for managing arterial hypertension. This strategy is designed to achieve long-term blood pressure control, reducing the risk of complications associated with chronic arterial hypertension, including recurrent intracranial hemorrhage and cardiovascular events. Surgical intervention was deemed unnecessary, as there were no signs of elevated intracranial pressure. At least once daily physiotherapy, occupational therapy, and speech therapy were provided throughout the 8-day hospital stay. By the time of discharge, the patient's vertigo, trunk tilt, and resulting gait instability had resolved, though minimal Grade I gaze-evoked nystagmus persisted.

## 3. Discussion

This patient had pseudovestibular syndrome due to a left paramedian cerebellar hemorrhage, which mimicked right-sided acute peripheral vestibulopathy. Cerebellar hemorrhage rarely causes an isolated vestibular syndrome, since it is more often associated with headache, ataxia, or—in case of mass effect —symptoms of increased intracranial pressure [8]. However, it has been previously suggested as a possible cause of pseudovestibular syndrome [1], but to our knowledge, this is the first detailed report of this condition. Similar to pseudovestibular syndrome following cerebellar infarction, damage to the connective fibers disrupts inhibitory inputs from the cerebellar flocculus to the ipsilateral VN in the lateral pons. This causes an imbalance in the vestibular system with increased activity in the ipsilateral VN, more specifically the medial (M) VN, resulting in horizontal nystagmus directed toward the ipsilateral side (Figure 1(c)).

Notably, while the combination of vertigo and spontaneous horizontal nystagmus, alongside a normal HIT, strongly suggests a central etiology, an abnormal HIT alone is not entirely reliable for diagnosing peripheral vestibulopathy due to its limited sensitivity. For instance, a study by Kim et al. reported a sensitivity of approximately 80% [9]. Consequently, clinical assessment should extend beyond the HIT and may include well-established diagnostic techniques such as HINTS, HINTS Plus (which incorporates an additional assessment of auditory function) [10], and other evaluations such as head-shaking nystagmus, direction-changing nystagmus with fixation, saccade hypermetria (clearly indicating cerebellar etiology), or (central) positional nystagmus. This is a limitation of this case report, as results on these additional examinations were not available. The results of the vHIT—distinct VOR gain reduction on the right and a less pronounced reduction on the left—might suggest right-sided vestibulopathy. However, as described above, central lesions affecting the flocculus can lead to a pathological VOR, and central lesions often result in bilaterally positive HITs. A recent study by Kim et al. found that in patients with posterior circulation strokes, about 54% had normal HIT results, while 19% showed ipsilaterally positive results, 17% bilaterally positive results, and 10% contralaterally positive results [9]. The contralateral reduction in VOR gain—typically marginal in cases of peripheral vestibulopathy—may be explained by compensation from the healthy side after acute unilateral vestibular dysfunction.

Additionally, the patient had a history of head and neck carcinoma, with prior surgery on the right parotid and radiation therapy at the tumor site. This may have contributed to the observed dysfunction of the right vestibular apparatus [11], providing a possible explanation for the findings on vHIT.

This case may help raise awareness of the so-called pseudovestibular syndrome and emphasize the importance of supplementary imaging diagnostics when the medical history or clinical presentation raises doubts about the peripheral origin of an apparent vestibular syndrome. It also highlights that, even when clinical presentation and pathological VOR suggest a peripheral origin, a central etiology can be a differential diagnosis.

## Data Availability

The data that support the findings of this study are available on request from the corresponding author. The data are not publicly available due to privacy or ethical restrictions.
